# N-Glycan Branching Affects the Subcellular Distribution of and Inhibition of Matriptase by HAI-2/Placental Bikunin

**DOI:** 10.1371/journal.pone.0132163

**Published:** 2015-07-14

**Authors:** Ying-Jung J. Lai, Hsiang-Hua D. Chang, Hongyu Lai, Yuan Xu, Frank Shiao, Nanxi Huang, Linpei Li, Ming-Shyue Lee, Michael D. Johnson, Jehng-Kang Wang, Chen-Yong Lin

**Affiliations:** 1 Lombardi Comprehensive Cancer Center, Department of Oncology, Georgetown University, Washington DC, United States of America; 2 Department of Biochemistry, National Defense Medical Center, Taipei City, Taiwan; 3 Affiliated Hospital of Hunan Traditional Chinese Medicine Research Institute, Changsha, Hunan, China,s; 4 Graduate Institute of Biochemistry and Molecular Biology, College of Medicine National Taiwan University, Taipei City, Taiwan; Hormel Institute, University of Minnesota, UNITED STATES

## Abstract

The gene product of *SPINT 2*, that encodes a transmembrane, Kunitz-type serine protease inhibitor independently designated as HAI-2 or placenta bikunin (PB), is involved in regulation of sodium absorption in human gastrointestinal track. Here, we show that *SPINT 2* is expressed as two species of different size (30-40- versus 25-kDa) due to different N-glycans on Asn-57. The N-glycan on 25-kDa HAI-2 appears to be of the oligomannose type and that on 30-40-kDa HAI-2 to be of complex type with extensive terminal N-acetylglucosamine branching. The two different types of N-glycan differentially mask two epitopes on HAI-2 polypeptide, recognized by two different HAI-2 mAbs. The 30-40-kDa form may be mature HAI-2, and is primarily localized in vesicles/granules. The 25-kDa form is likely immature HAI-2, that remains in the endoplasmic reticulum (ER) in the perinuclear regions of mammary epithelial cells. The two different N-glycans could, therefore, represent different maturation stages of N-glycosylation with the 25-kDa likely a precursor of the 30-40-kDa HAI-2, with the ratio of their levels roughly similar among a variety of cells. In breast cancer cells, a significant amount of the 30-40-kDa HAI-2 can translocate to and inhibit matriptase on the cell surface, followed by shedding of the matriptase-HAI-2 complex. The 25-kDa HAI-2 appears to have also exited the ER/Golgi, being localized at the cytoplasmic face of the plasma membrane of breast cancer cells. While the 25-kDa HAI-2 was also detected at the extracellular face of plasma membrane at very low levels it appears to have no role in matriptase inhibition probably due to its paucity on the cell surface. Our study reveals that N-glycan branching regulates HAI-2 through different subcellular distribution and subsequently access to different target proteases.

## Introduction

Hepatocyte growth factor activator inhibitor (HAI)-2/placental bikunin (PB) is an integral membrane, Kunitz-type serine protease inhibitor [1,2]. HAI-2 was initially identified and purified from the conditioned medium of MNK 45 human gastric cancer cells based on its ability to inhibit the serine protease hepatocyte growth factor activator (HGFA) from which it received its name [1]. HAI-2 mutations have been shown to cause the syndromic form of congenital sodium diarrhea (CSD) [3,4]. CSD is an autosomal recessive disorder of sodium/proton exchange, characterized by postnatal watery diarrhea with high fecal sodium excretion [5]. This suggests that HAI-2 may be involved in the regulation of sodium exchange in the gastro-intestinal (GI) track. One of the mutations, Y163C, is localized within the second Kunitz domain and results in reduced inhibitory activity of HAI-2 against trypsin. The reduction or loss of HAI-2 protease inhibitory activity that this mutation produces may result in the excessive proteolytic activity of a protease(s) normally inhibited by HAI-2 and that has some role in regulating sodium exchange. Indeed, in *Xenopus laevis* oocytes, HAI-2 can inhibit the function of several membrane-bound serine proteases that alter the activity of an epithelial sodium channel (ENaC) [6]. As appealing as the hypothesis that HAI-2 regulates sodium exchange via its target proteases on the cell surface may be, the localization of HAI-2 is predominantly intracellular *in vivo* in human colon epithelial cells [7,8] and in cultured human mammary epithelial cells [9]. This suggests that HAI-2-mediated proteolysis control is likely to be an intracellular event, which is at odds with the hypothesis that HAI-2 regulates sodium absorption on the apical surface of colon epithelial cells, through which sodium ions are absorbed from the lumen of the colon. Alternatively, for a role in sodium absorption HAI-2 might require translocation to the cell surface in a regulated rather than a constitutive fashion. The hypothesis is supported by the occasional detection of HAI-2 on the apical surface plasma and in the lumens of the human colon mucosa epithelium [7].

The identification of the HAI-2 target proteases involved in the control of sodium exchange is also not simple. Several serine proteases can be inhibited by HAI-2 in solution, but it is not clear whether this *in vitro* inhibitory activity is relevant to the regulation of sodium exchange *in vivo*. For example, HAI-2 was given its name based on its initial identification as an inhibitor of HGFA, a secreted and blood-borne serine protease predominantly of hepatocyte origin [10,11]. It does not seem likely that HAI-2 regulation of HGFA activity has any relation to the regulation of sodium exchange at the apical surface of colon epithelial cells. Mouse model studies appear to add more complexity to the identification of the HAI-2 target proteases involved in the regulation of sodium exchange. Mouse matriptase, a type 2 transmembrane serine protease, has been identified as a HAI-2 target protease by virtue of the fact that defects in placental development caused by targeted deletion of HAI-2 in mouse can be rescued by simultaneous deletion of matriptase [12]. Human matriptase is, however, not likely to be a relevant HAI-2 target protease involved in sodium exchange in the GI track for two reasons. Firstly, HAI-2 inhibits matriptase via the first of its two Kunitz domains (N-terminal) but not the second (C-terminal) domain and so the HAI-2 Y163C mutation found in CSD patients would not be expected to impact the inhibitory activity of HAI-2 against matriptase since it is located in the second Kunitz domain. Secondly, matriptase is predominantly expressed at cell-cell junctions whereas HAI-2 is primarily detected in vesicle-like structures, largely inside cells. These different subcellular localizations make it less likely that matriptase is the major HAI-2 target protease involved in sodium exchange. Although HAI-2 is translocated to the surface of breast cancer cells, where HAI-2 then gains access to and can inhibit matriptase, the function of HAI-2-mediated matriptase inhibition remains to be determined.

HAI-2 contains two putative N-glycosylation sites and is highly glycosylated. N-glycans contribute approximately 50% of the molecular mass of the HAI-2 secreted by MKN45 gastric cancer cells [1]. In addition to the challenge of identifying the relevant HAI-2 target proteases and the poor understanding of the molecular mechanism by which HAI-2 contributes to the regulation of sodium exchange, the nature and role of N-glycosylation in the anti-protease activity and pathophysiological function of HAI-2 have been not explored. In the current study, HAI-2 was shown to be expressed with two different types of N-glycan: with or without extensive N-glycan branching. These two types of N-glycan likely result from the different stages of HAI-2 N-glycosylation and alter the exposure of HAI-2 epitopes, HAI-2 subcellular localization, and the access of HAI-2 to target proteases, such as matriptase.

## Materials and Methods

### Chemicals and reagents

Wheat germ agglutinin (WGA)-Agarose and Concanavalin (Con) A-Agarose were obtained from Vector Labs (Burlingame, CA). 5,5’-Dithio-bis-(2-Nitrobenzoic Acid) (DTNB), 4',6-diamidino-2-phenylindole (DAPI), and tunicamycin were obtained from Sigma-Aldrich (St. Louis, MO); Peptide N-glycosidase F (PNGase) was purchase from New England Biolabs (Ipswich, MA); Swainsonine was purchased from Cayman Chemical (Ann Arbor, MI). Soluble recombinant HAI-2 was purchased from Novoprotein (Summit NJ). CNBr-activated Sepharose 4B was obtained from GE Healthcare (Uppsala, Sweden). Alexa Fluor 488 Phalloidin, Alexa Fluor 594 goat anti-mouse IgG, and an Alexa Fluor 488 Antibody Labeling kit were obtained from Life Technologies (Lifetechnologies.com). Fluoro-Gel with TES Buffer was obtained from Electron Microscopy Sciences (Hatfield, PA). ProtoBlue Safe stain was obtained from National Diagnostics (Atlanta, GA).

### Cell lines and cell cultures

The culture medium and conditions for the cell lines used in the study have been described in our recent publication [9]. The sources of these cells were as follows: 184 A1N4 human mammary epithelial cells were a gift from M. R. Stampfer, (UC Berkeley) [13]; HaCaT human keratinocytes were obtained from Cell Lines Service GmbH (Eppelheim Germany); the human head and neck squamous carcinoma cells SCC-25/CP [14] and PCI-51 [15,16] were generated at the University of Pittsburgh; JHU-011 and JHU-028 were developed at Johns Hopkins University; the rest of cell lines were obtained from American Type Culture Collection (ATCC). These cells included the human breast cancer cells, MCF-7, T-47D, MDA-MB-231 and SK-BR-3, RWPE-1 human prostate epithelial cells, LNCaP, CWR22, DU145, and PC3 prostate cancer cells, the pancreatic adenocarcinoma cell line Colo 357, the human placental choriocarcinoma cell line JEG-3, the human lung carcinoma cell line A549, the human hepatocellular carcinoma cell line Hep G2 and the human colon carcinoma cell lines, Caco2, HT-29, and LS 174T.

### Antibodies

The HAI-2 mAb DC16 [9] and XY9 were generated by hybridoma fusion of cells isolated from the spleen of a mouse immunized with soluble recombinant HAI-2 (Novoprotein, Summit NJ) as the previously described [9]. The mouse monoclonal antibodies M24, M69, and M19 were used for immunoblot analyses to detect total matriptase, activated matriptase and HAI-1, respectively as we have previously described [17–19]. The HAI-1 mAb M19 and HAI-2 mAb DC16 were immobilized on Sepharose 4B at 5mg/ml beads, using CNBr-activated beads, following the manufacturer’s instruction and were used for the immunodepletion experiments. The rabbit polyclonal antibody directed against protein disulfide isomerase was obtained from Stressgen (Catalog number SPA-890, Victoria, BC, Canada). FITC-labelled mouse anti-GM130 monoclonal antibody was purchased from BD Bioscience Pharmingen (Catalog number 612009).

### Protein pull-down by lectin-Agarose and mAb-Sepharose

For immunodepletion, protein samples (200 μl) were mixed with 15 μl mAb-Sepharose 4B beads. For Lectin pull-down, protein samples (100 μl) were mixed with 50 μl lectin-Agarose beads. The mixtures were rotated in a cold room for 2 hours. The supernatant containing the unbound proteins was separated from the agarose beads by centrifugation and collected.

### Deglycosylation of HAI-2 by PNGase

Fifty μg soluble recombinant HAI-2 (Novoprotein, Summit NJ) in 0.5% SDS and 40 mM DTT was denatured by heating at 100°C for 10 min. The sample was adjusted to the reaction conditions containing 0.05 M sodium phosphate and 1% NP-40. PNGase F (500 U) was added, and the mixture was then incubated at 37°C for 1 hour. The reaction was terminated by boiling the samples in SDS sample buffer containing DTT for 5 min, followed by SDS-PAGE and immunoblot analysis. Control samples were treated in the same manner except for the addition of the PNGase F.

### Western blotting

Cell lysates were prepared by lyzing the cells with 1% Triton X-100 and 1 mM DTNB in phosphate buffered saline (PBS). DTNB was added to the lysis buffer to prevent the cleavage of a disulfide linkage which holds together the serine protease domain and the non-catalytic domains of activated matriptase [20]. Lysate protein concentrations were determined by Bradford protein assay. Conditioned medium prepared by culturing MCF 7 breast cancer cells in serum-free culture medium for 24 hours was collected and concentrated by approximately 100-fold using Millipore Amicon Centrifugal Filter Devices. Equal amounts of proteins or equal proportions of cell lysates and conditioned medium were analyzed by Western blot. Protein samples were diluted with 5x SDS sample buffer containing no reducing agent and incubated at room temperature for 5 min. Proteins were resolved by 7.5% SDS-PAGE, transferred to nitrocellulose membranes, and probed with the indicated mAbs, followed by HRP conjugated secondary antibodies. The signals were visualized using the Western Lightening^®^ Chemiluminescence Reagent Plus (Perkin-Elmer, Boston, MA).

### Immunofluorescence

Human mammary epithelial cells (184 A1N4) and T-47D human breast cancer cells were cultured on 18 mm circular cover slides in 12-well plate. 184 A1N4 mammary epithelial cells were stimulated with fresh culture medium for 30 min [21,22] before being fixed in 10% buffered formalin (Fisher Scientific) for 20 min. To induce matriptase zymogen activation the T-47D breast cancer cells were treated with 150 mM phosphate buffer pH 6.0 for 20 min. The cells were then either permeabilized or not permeabilized using 0.5% Triton in PBS buffer for 5 min after being fixed in 10% buffered formalin. For studies to examine the co-localization of the 25-kDa HAI-2 form with protein disulfide isomerase, 184 A1N4 were incubated with the HAI-2 mAb XY9 at 2 μg/ml and an anti-protein disulfide isomerase rabbit polyclonal antibody (1:500) at room temperature for 60 min followed by staining with Alexa Fluor 488 goat anti-mouse IgG and Alexa Fluor 594 goat anti-rabbit IgG for 60 min. DAPI staining of the nucleus was used as a counterstain. To examine the co-localization of the 30-40-kDa HAI-2 species with the cis-Golgi protein GM-130, 184 A1N4 cells were incubated with the HAI-2 mAb DC16 at 2 μg/ml for 60 min, followed by Alexa Fluor 594 goat anti-mouse IgG, after which the cells were incubated with FITC-labeled anti-human GM130 mouse mAb (5 μg/ml) in PBS containing 3% BSA and 250 μg/ml mouse IgG for 60 min. To examine the co-localization of the 30-40-kDa and 25-kDa forms of HAI-2, or with activated matriptase in T-47D breast cancer cells, the cells were firstly stained with unlabeled HAI-2 mAb XY 9 (2 μg/ml) or activated matriptase mAb M69 (2 μg/ml) followed by Alexa Fluor 555-labled goat anti-mouse IgG. The cells were then washed extensively and stained with Alexa Fluor 488-conjugated HAI-2 mAb DC16 (5 μg/ml) in a solution containing 0.5 mg/ml mouse IgG for 30 min. Images were captured using a Zeiss LSM 510 confocal microscope.

## Results

### Endogenously HAI-2 is detected in immunoblot assays as three bands, which can be distinguished using two different HAI-2 mAbs

**Fig 1:**
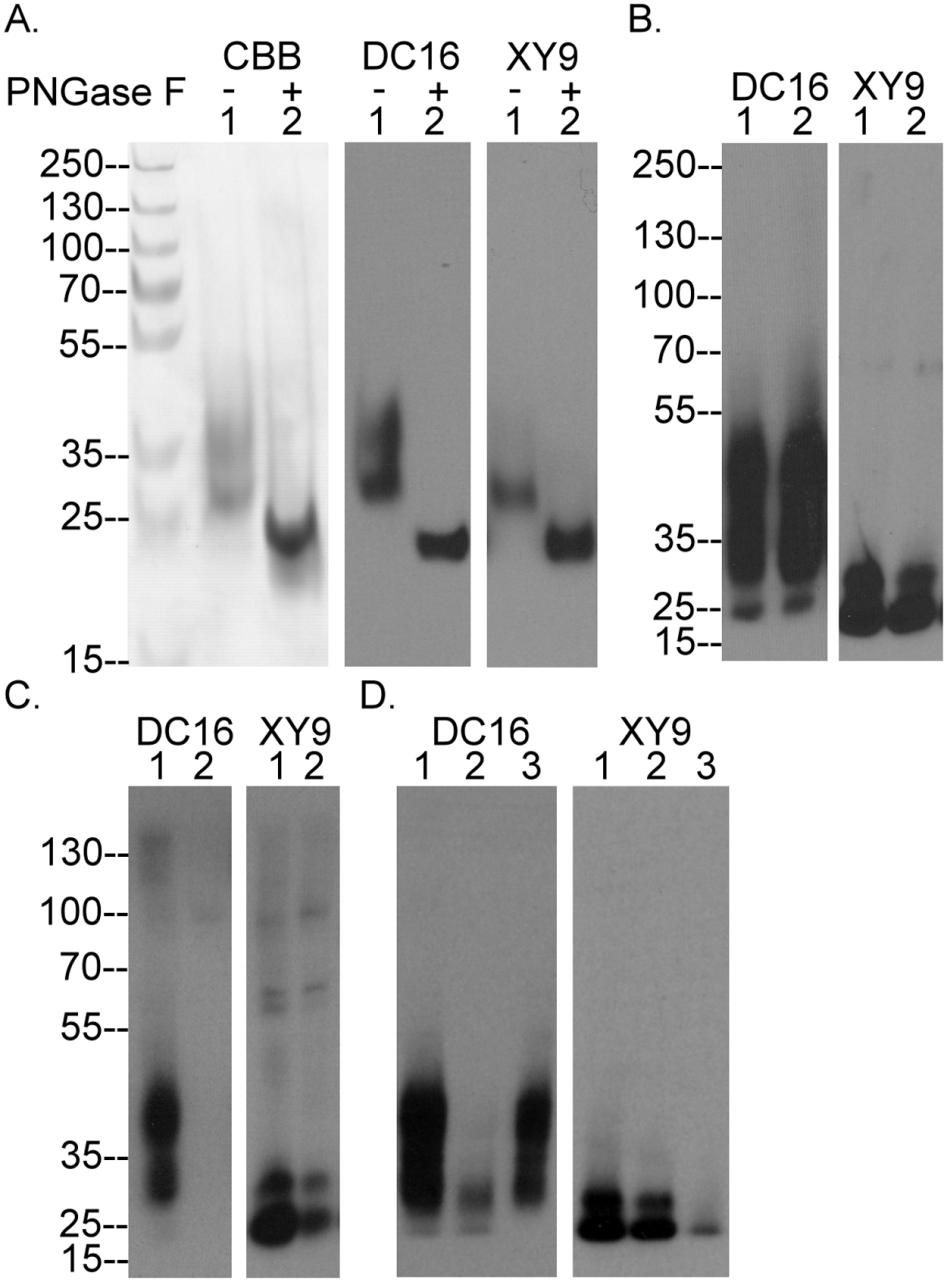
Defining HAI-2 species with two distinct mAbs. (A) Soluble recombinant HAI-2 was treated without (lanes 1) or with PNGase F (lanes 2) to remove N-glycans. The sizes of the HAI-2 preparations were analyzed by SDS-PAGE and visualized by Coomassie Brilliant Blue (CBB) staining. The immunoreactivity of the HAI-2 preparations was analyzed by Western blot using the mAbs DC16 and XY9. The sizes of molecular markers were as indicated. (B) Naturally occurring HAI-2 from 184 A1N4 (lanes 1) and MTSV 1.7 (lanes 2) human mammary epithelial cells was analyzed by immunoblot using the mAbs DC16 and XY9. (C) Cell lysates prepared from184 A1N4 cells were subjected to immunodepletion using the mAb DC16 linked to beads. The cell lysates prior to (lanes 1) and after immunodepletion (lanes 2) were analyzed by immunoblot using the mAb DC16 and XY9. (D) Cell lysates prepared from 184 A1N4 cells were incubated with WGA-Agarose or Con A-Agarose to deplete glycoproteins. The original lysate (lanes 1), WGA-depleted lysate (lanes 2), and Con A-depleted lysate (lanes 3) were analyzed by immunoblot using the mAbs DC16 and XY9.

Naturally occurring HAI-2 isolated from the conditioned medium of human gastric cancer cells contains significant amounts of N-linked glycan, which increases the apparent molecular weight by the addition of 9-17-kDa to the mass of the unmodified HAI-2 polypeptide of 14-kDa, as determined by SDS PAGE [1]. The soluble recombinant human HAI-2 protein (Ala28-Lys197 plus Val-Asp-6xHis) used in this study was produced in HEK-293 cells (Novoprotein Scientific Inc., Summit NJ) and resembles the naturally occurring HAI-2 with respect to the level of N-glycosylation and appears as two bands on SDS gels stained with Coomassie G250 (CBB): an upper band with a more diffuse appearance and an apparent size of 35-kDa, and a lower band of more compact appearance with a size of 27-kDa (Fig 1A, CBB, lane 1). Removal of the N-glycan from the HAI-2 preparation with Peptide: N-Glycosidase F (PNGase F) resulted in a 23-kDa protein band (Fig 1A, CBB, lane 2). Western blot analysis using the mAb DC16 (Fig 1A, DC16) revealed a similar HAI-2 staining profile to that seen by CBB staining before and after the removal of the N-glycan, confirming the ability of this antibody to detect HAI-2. The detection of HAI-2 following the removal of N-glycan suggests that the epitope recognized by the DC16 mAb resides on the HAI-2 polypeptide. In addition to the DC16 mAb, we also generated another HAI-2 mAb, named XY9. The XY9 mAb can detect the HAI-2 polypeptide after PNGase F treatment (Fig 1A, XY9 lane 2) and also the lower band of N-glycosylated HAI-2, but not the upper band (Fig 1A, XY9 lane 1), suggesting that the epitope recognized by the XY9 mAb also resides on the HAI-2 polypeptide but that it is likely masked by extensive N-glycosylation of HAI-2.

A survey of a variety of different human cell cultures, both in our previous [9] and in the current study using the two HAI-2 mAbs, led us to the conclusion that human epithelial and carcinoma cells express HAI-2 in multiple forms yielding three bands detected in immunoblots with apparent sizes of 25-, 30- and 40-kDa (Fig 1B), which are detected differentially by the two HAI-2 mAbs. The mAb DC16 can readily detect the 30- and 40-kDa HAI-2 bands, but barely detects the 25-kDa species, which can only be seen as a minor band only after long exposures in immunoblot analysis of the two human mammary epithelial cells, MTSV 1-1B and 184 A1N4 (Fig 1B, DC16). In contrast, the mAb XY9 can readily detect HAI-2, as a doublet of 25-kDa (Fig 1B, XY9). Immunodepletion of the lysates using the mAb DC16 completely removed the 40- and 30-kDa HAI-2 bands, as expected (Fig 1C, DC16, lane 2) but also significantly depleted the 25-kDa HAI-2 doublet, recognized by the XY9 mAb (Fig 1C, XY9, lane 2). This immunodepletion experiment supports the idea that the epitope recognized by the mAb DC16 is present, but largely masked, on the 25-kDa HAI-2 band (Fig 1C). Collectively, these data suggest that HAI-2 can be expressed by cells with different degrees of N-glycosylation, which appears to influence the exposure of the two epitopes recognized by the two HAI-2 mAbs.

### Differential N-glycan branching of the different HAI-2 species

The observation that HAI-2 is produced by cells with several distinct levels of N-glycosylation suggested the possibility that there are defined differences in the structures of the N-glycan involved. To examine this possibility we tested the ability of the various HAI-2 species to bind to the lectins, concanavalin A (Con A) and wheat germ agglutinin (WGA). The 30- and 40-kDa HAI-2 forms bound to and were depleted from the lysate by WGA-Agarose (Fig 1D, DC16, lane 2) but these HAI-2 species did not bind to Con A-Agarose and were left in the unbound fraction (Fig 1D, DC16, lane 3). In contrast, the 25-kDa HAI-2 form did not bind to WGA-Agarose and remained in the unbound fraction (Fig 1D, XY9, lane 2), but did bind to Con A-Agarose and so was significantly depleted by those beads (Fig 1D, XY9, lane 3). These data suggest that the N-glycan on the 25-kDa HAI-2 form possesses exposed terminal mannose residues but no N-acetyl glucosamine, whereas the N-glycan found on the 30- and 40-kDa HAI-2 forms has exposed terminal N-acetyl glucosamine but no mannose. The size and the differential lectin binding suggest that the N-glycan on the 25-kDa HAI-2 is likely of the oligomannose type and without N-glycan branching. In contrast, the 30- and 40-kDa forms of HAI-2 are likely modified with complex type N-glycan with extensive N-glycan branching by addition of N-acetyl glucosamine.

**Fig 2:**
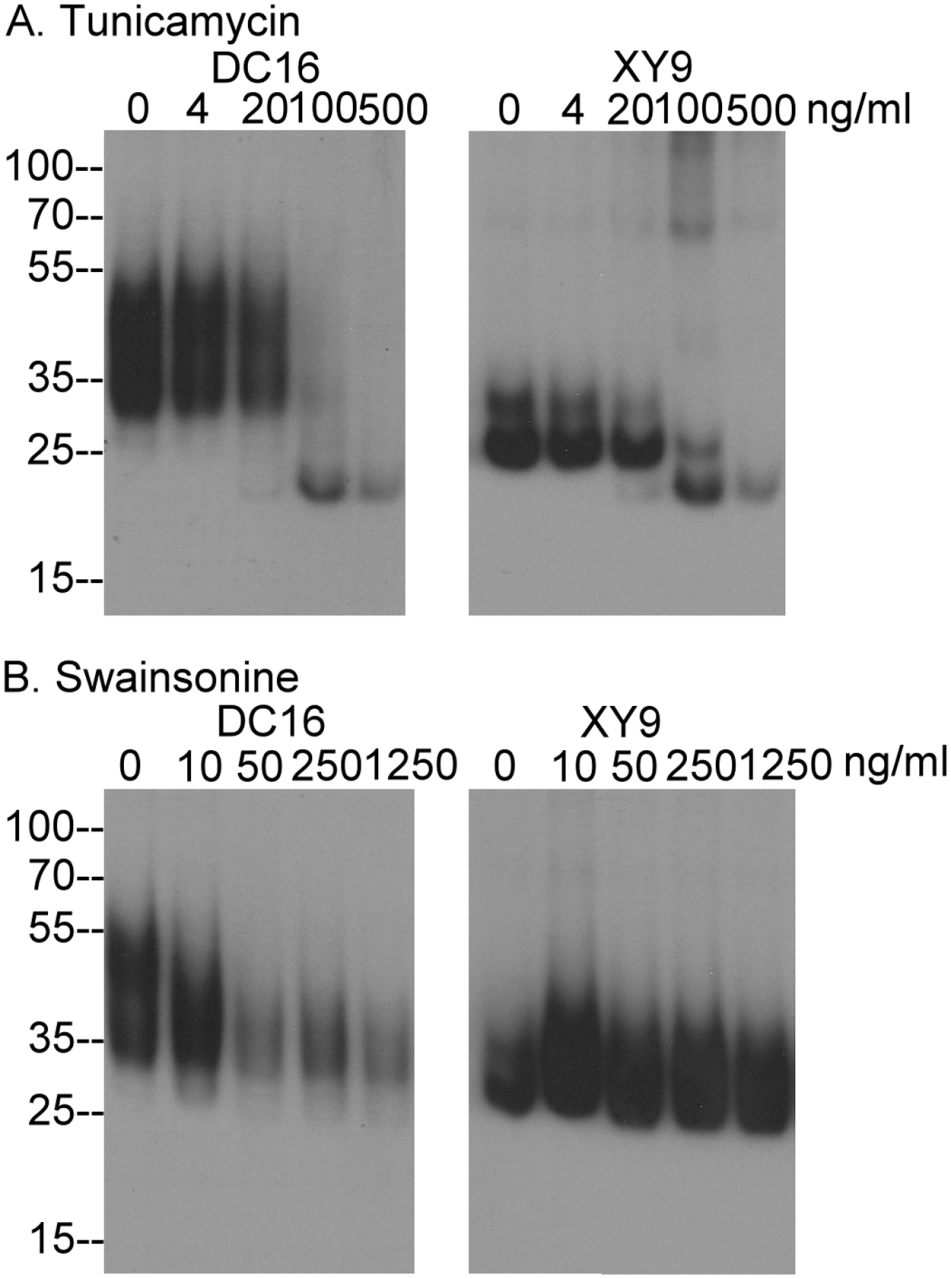
N-glycosylation and N-glycan branching of HAI-2. Human mammary epithelial cells 184 A1N4 were treated with tunicamycin (A) or swainsonine (B) overnight at the indicated concentrations. The cell lysates were analyzed by immunoblot for HAI-2 species using the mAbs DC16 and XY9.

We next determined how N-glycosylation and N-glycan branching contribute to the different extent of N-glycosylation of HAI-2 and the exposure of the epitopes recognized by the two HAI-2 mAbs. Protein N-glycosylation initially occurs by the transfer of a 14 sugar glycan to the nascent polypeptide during translation in the endoplasmic reticulum, a process that can be inhibited by tunicamycin [23]. Treatment of 184 A1N4 human mammary epithelial cells with tunicamycin at a concentration of 100 ng/ml resulted in the disappearance of both the 30- and 40-kDa HAI-2 forms (Fig 2A, DC16) and also the 25-kDa HAI-2 doublet (Fig 2A, XY9). Concurrent with the disappearance of these three HAI-2 forms was the appearance of a 23-kDa HAI-2 band (Fig 2A), which is likely the HAI-2 polypeptide having a size close to that of the calculated mass (Fig 2A). Furthermore, detection of the HAI-2 polypeptide by both HAI-2 mAbs suggests that the epitopes recognized by the antibodies reside on the HAI-2 polypeptide and not the N-glycan. This result is in good agreement with the situation observed for the recombinant HAI-2 shown in Fig 1, in which removal of N-glycan from soluble recombinant HAI-2 with PNGase F resulted in the generation of a HAI-2 species with the size of the polypeptide and the exposure of the epitopes recognized by the both HAI-2 mAbs (Fig 1A).

Following the initial N-glycosylation in the ER, addition, removal and modifications of N-glycan subsequently takes place in the Golgi apparatus. Inhibition of α-mannosidase II by swainsonine at a concentration of 50 ng/ml prevented N-glycan branching, and significantly reduced the levels of the 30- and 40-kDa HAI-2 bands (Fig 2B, DC16), suggesting a role for of N-glycan branching in the generation of those HAI-2 species. In contrast, the level of the 25-kDa HAI-2 form is significantly increased by the suppression of α-mannosidase II activity with swainsonine even at a concentration of 10 ng/ml, at which the level of the 30- and 40 kDa HAI-2 forms were not significantly reduced (Fig 2B, XY9). These data suggest that the 25-kDa HAI-2 form does not undergo N-glycan branching, consistent with its binding to Con A but not WGA. Thus, HAI-2 can be expressed by cells with and without N-glycan branching.

**Fig 3:**
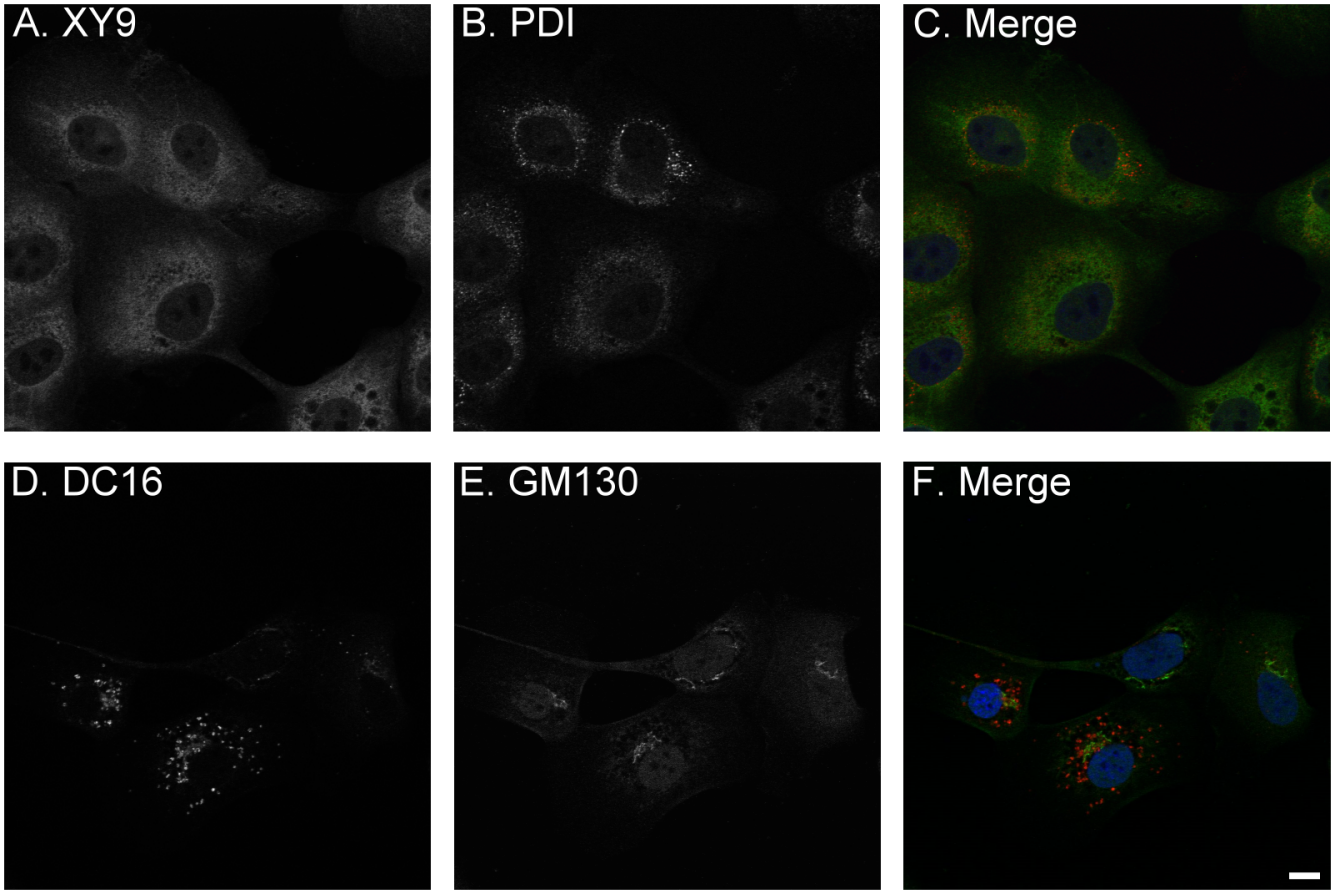
Subcellular localization of HAI-2 species in human mammary epithelial cells. The subcellular localizations of 25-kDa HAI-2 (A and C, green), compared with protein disulfide isomerase (PDI), an ER marker (B, and C, red) and 30-40-kDa HAI-2 (D and F, red), compared with GM130, a cis-Golgi marker (E and F, green) were analyzed by immunofluorescent double staining. The cells were also stained for nuclei using DAPI (blue), as a counterstain. The staining is presented as black and white images (A, B, D, and E) or merged false-color images (C and F). Scale bar: 10 μm.

The increase in the level of the 25-kDa HAI-2 by blockade of N-glycan branching and the decrease in the levels of the 40- and 30-kDa HAI-2 suggests that a proportion of the 25-kDa HAI-2 may be converted to the 30- and 40-kDa HAI-2 via N-glycan branching and that, therefore, the 25-kDa HAI-2 may serve as a precursor to the 30- and 40-kDa HAI-2. This hypothesis is supported by the subcellular localization of the HAI-2 species (Fig 3). Immunofluorescence staining of 184 A1N4 mammary epithelial cells for the 25-kDa form of HAI-2 and protein disulfide isomerase, a marker for the endoplasmic reticulum (ER), revealed that the 25-kDa form of HAI-2 is likely localized within the ER (Fig 3, XY 9). In contrast, the 30- and 40-kDa HAI-2 forms were detected by the DC16 mAb primarily in vesicles/granules a few of which also coincided with staining for GM130, a cis-Golgi marker (Fig 3). These data suggest that the HAI-2 species recognized by the mAb DC16 have been processed through the Golgi apparatus with the vast majority having completed their N-glycan branching and exited the Golgi apparatus into secretory vesicles/granules.

**Fig 4:**
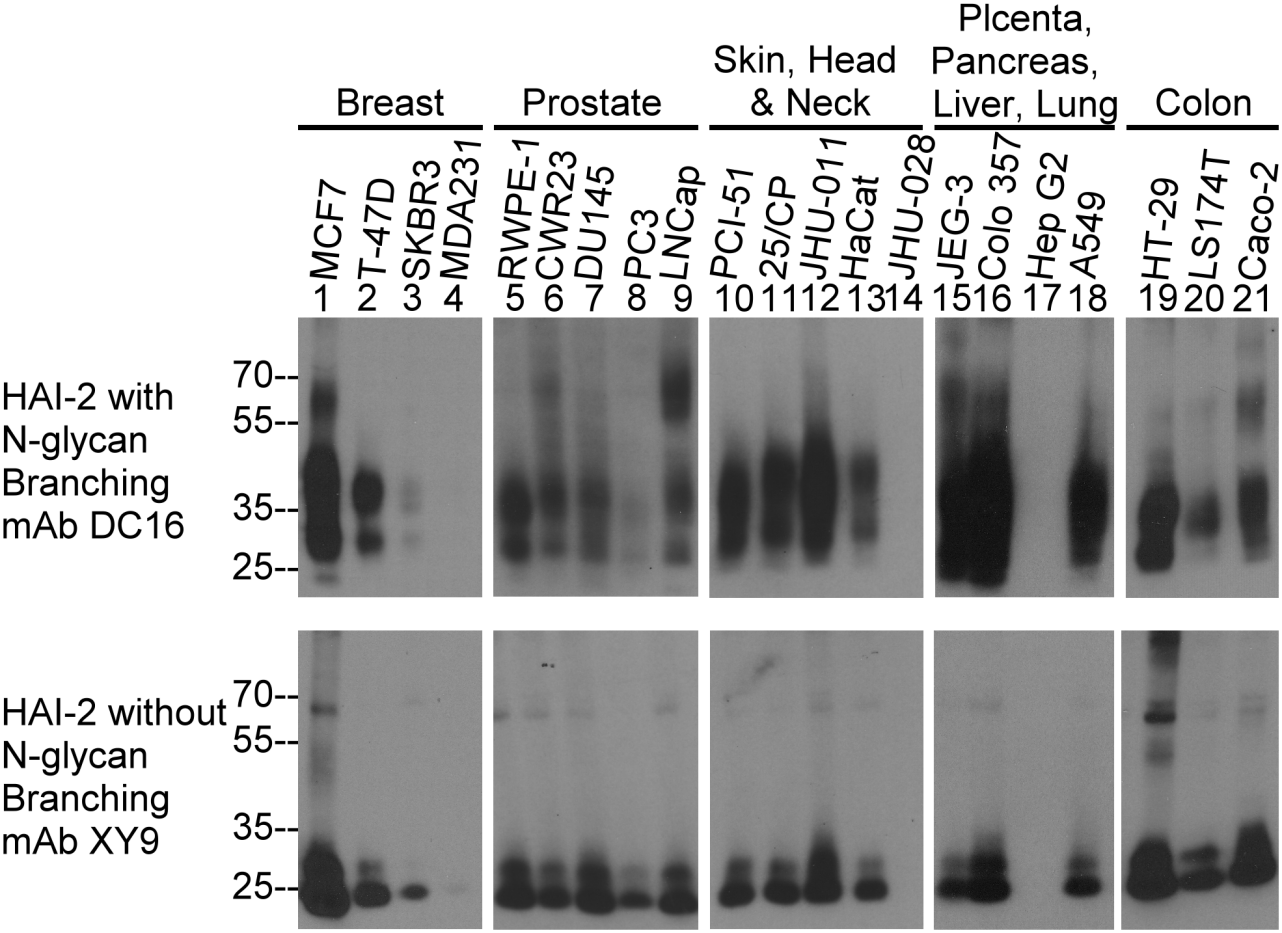
Expression of HAI-2 species in human epithelial and carcinoma cells. Cell lysates were prepared from 21 human epithelial and carcinoma lines derived from the organ systems indicated. The expression of HAI-2 species with and without N-glycan branching was analyzed by immunoblot using the HAI-2 mAbs DC16 (upper panels) and XY9 (low panels). Equal concentrations of total cellular protein from each cell line within each organ system were analyzed.

The relative expression of the differentially glycosylated forms of HAI-2 was then analyzed in a variety of human epithelial and carcinoma cells by immunoblot analysis of cell lysates using the two HAI-2 mAbs (Fig 4). Among the 21 human cell lines, the 19 lines that were mAb DC16 positive were also positive for the XY9 mAb. The 2 lines that were mAb DC16 negative were also negative for the mAb XY9 (Fig 4, lanes 14 and 17), suggesting that the two HAI-2 species are completely co-expressed among different cells. Furthermore, there was a general trend that those cells with high levels of the 30- and 40-kDa HAI-2 forms also had high levels of 25-kDa HAI-2 and vice versa. Thus, although HAI-2 is expressed at quite different levels by the breast cancer cell lines MCF7, T-47D, SKBR3, and MDA MB 231, the ratio between the HAI-2 species recognized by the two antibodies remained similar (Fig 4, lanes 1–4). It should be noted that the levels of HAI-2 species in MDA MB 231 was very low compared to that of T-47D and MCF7 and required a much longer exposure time for the immunoblot assay to detect the signals (Fig 4, lanes 4). These data suggest that in the vast majority of epithelial and carcinoma cells, HAI-2 is expressed both with and without N-glycan branching. Interestingly, the DC16 HAI-2 antibody recognized additional bands at higher molecular weights in some cell lines that may represent HAI-2-containing complexes. For example, HAI-2 species with N-glycan branching were detected in bands at 60-kDa in MCF7, LNCaP, and Caco-2 cells (Fig 4, lanes 1, 9, and 21).

### The 40-and 30-kDa but not the 25-kDa form of HAI-2 inhibits matriptase in breast cancer cells

**Fig 5:**
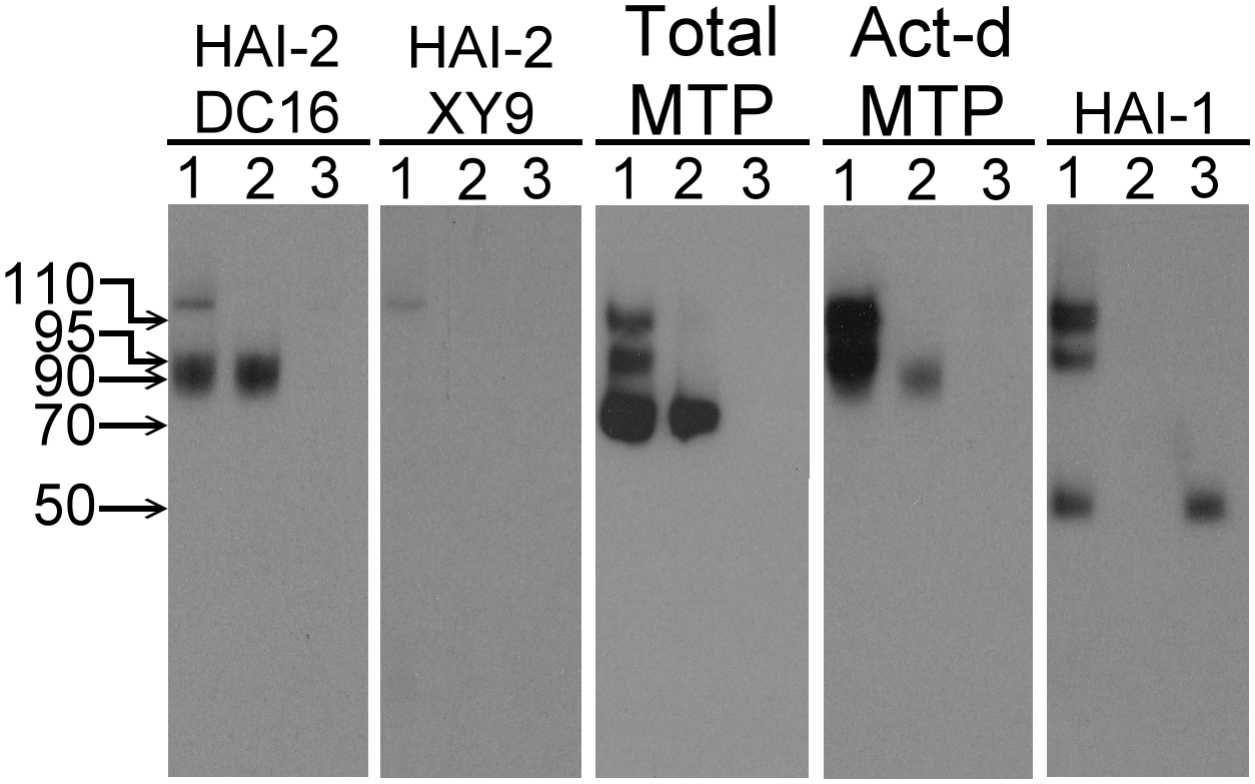
Activated matriptase is shed in complexes with HAI-1 and the HAI-2 species with N-glycan branching. Conditioned medium from MCF7 breast cancer cells was collected and subjected to immunodepletion using HAI-1 mAb M19-Sepharose or matriptase mAb 21-9-Sepharose. The conditioned medium (lanes 1), HAI-1-depleted conditioned medium (lanes 2), and matriptase-depleted medium (lanes 3) were analyzed by immunoblot for HAI-2 species with N-glycan branching using the mAb DC16 (HAI-2 DC16), HAI-2 species without N-glycan branching using the mAb XY9 (HAI-2 XY9), total matriptase using the mAb M24 (Total MTP), activated matriptase using the mAb M69 (Act-d MTP), and HAI-1 using the mAb M19 (HAI-1).

Previously we have shown that HAI-2 can serve as an endogenous matriptase inhibitor in MCF7 and T-47D human breast cancer cells [9]. We therefore analyzed the HAI-2 species present in conditioned media from breast cancer cells to determine how the differentially glycosylated HAI-2 species contribute to matriptase inhibition. The mAb DC16 detected a 90-kDa protein band (Fig 5, DC16, lane 1), whereas the XY9 mAb did not detect anything with the exception of a minor protein band with a size of greater than 110-kDa, which is likely a non-specific protein band since it was also detected by other mAbs and even by the secondary antibody alone control (Data not shown). This non-specific band is most likely an Fc receptor, which also bound to the antibody beads (Fig 5, lanes 2 and 3). To confirm that the 90-kDa HAI-2 species also contained matriptase and to determine the extent to which HAI-2 contributes to matriptase inhibition relative to HAI-1, we conducted immunodepletion experiments with a HAI-1 mAb (Fig 5, lanes 2) and a matriptase mAb (Fig 5, lanes 3) using the conditioned medium (Fig 5B, lanes 1). The 90-kDa HAI-2 band was immunodepleted by the matriptase mAb (Fig 5, HAI-2 DC16, lane 3) but not by the HAI-1 mAb (Fig 5, HAI-2 DC16, lane 2), confirming that the 90-kDa HAI-2 band contains matriptase.

Regarding the assessment of the extent to which HAI-2 contributes to matriptase inhibition, matriptase was shed as three species of 70-, 95-, and 110-kDa (Fig 5, Total MTP, lane 1). The 95- and 110-kDa species were activated matriptase in complex with HAI-1, both of which were also detected by the activated matriptase mAb (Fig 5, Act-d. MTP, lane 1) and the HAI-1 mAb (Fig 5, HAI-1, lane 1), and were immunodepleted by the HAI-1 (Fig 5, lanes 2) and matriptase mAbs (Fig 5 lanes 3). The ratio of the 90-kDa matriptase-HAI-2 complex relative to the 110- and 95-kDa matriptase-HAI-1 complexes appeared to be quite low (Fig 5 Act-d. MTP, comparing lane 2 to lane 1). The low abundance of the matriptase-HAI-2 complex relative to the matriptase-HAI-1 complex makes it difficult to detect the matriptase-HAI-2 complex using the total matriptase mAb, even after immunodepletion of the matriptase-HAI-1 complexes from the sample (Fig 5, Total MTP, comparing lane 2 to lane 1). Collectively, these data suggest that in breast cancer cells matriptase is primarily under the control of HAI-1 and to minor extent by HAI-2 with N-glycan branched glycosylation. The HAI-2 species without N-glycan branching does not appears to have any role in matriptase inhibition.

### Differential plasma membrane translocation of HAI-2 species in breast cancer cells

**Fig 6:**
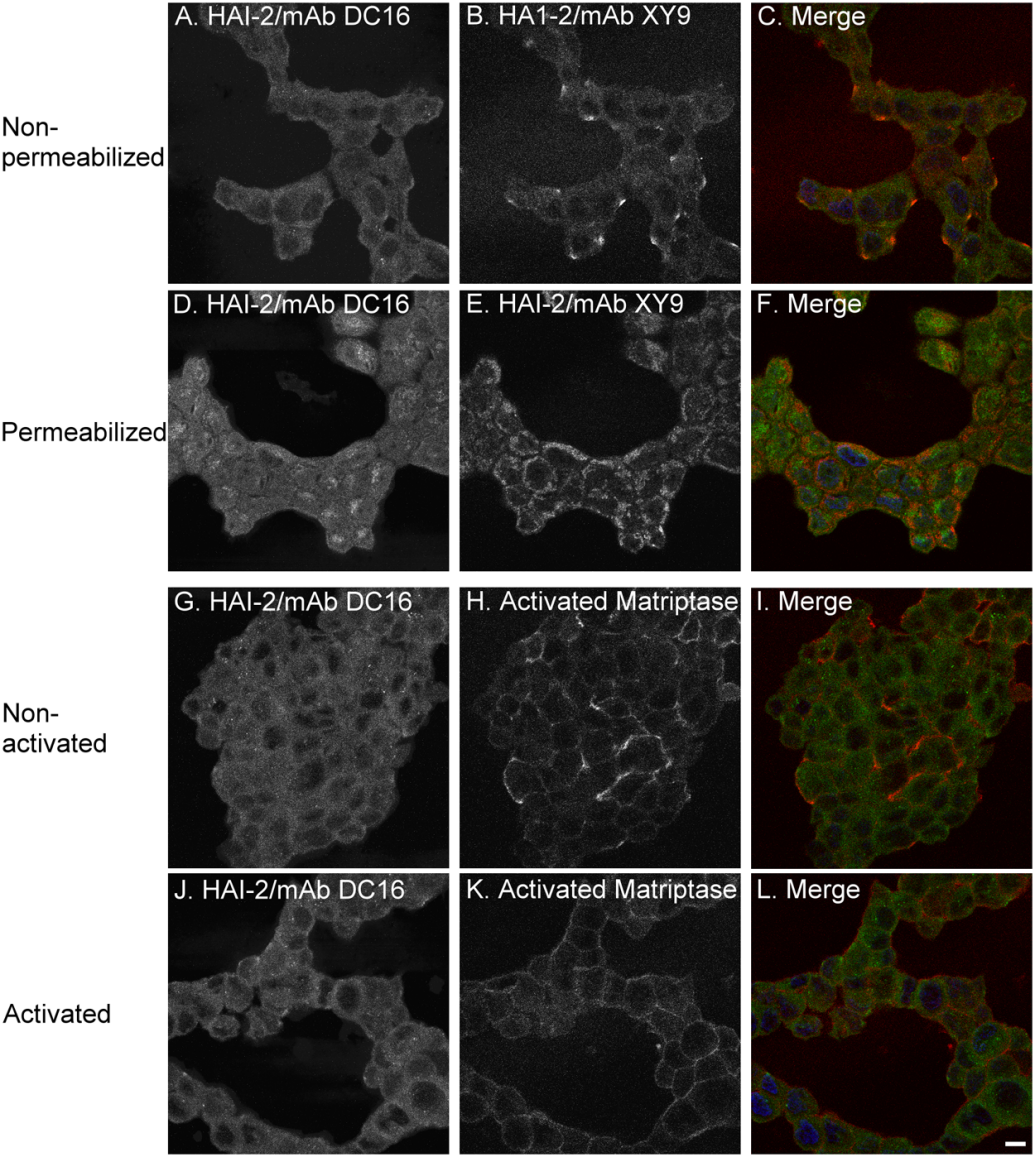
Subcellular localizations of HAI-2 species and activated matriptase in breast cancer cells. The subcellular localizations of HAI-2 species and activated matriptase in T-47D breast cancer cells were analyzed and compared by immunofluorescent double staining using Alexa 488-conjugated mAb DC 16 (green) with mAb XY9 (red) or the activated matriptase mAb M69 (red) after treatment of the cells with or without Triton X-100 to permeabilize the cells following the induction of matriptase zymogen activation by a pH 6.0 buffer exposure, as indicated. The cells were stained for nuclei using DAPI (blue), as a counterstain. The staining is presented as black and white images (A, B, D, E, G, H, J, and K) or merged false-color images (C, F, I and K). Scale bar: 10 μm.

The role of the different HAI-2 species in matriptase inhibition was further investigated by immunofluorescent double-staining of T47D breast cancer cells using the mAb DC16 conjugated with fluorescent dye Alexa-488 and the XY9 mAb (Fig 6). Under non-permeabilizing conditions, the HAI-2 species with N-glycan branching was detected by the DC16 mAb broadly on the cell surface (Fig 6A); in contrast, while the HAI-2 species without N-glycan branching was also detected on the cell surface, the level was very low and staining was focused on small areas of the plasma membrane of unknown identity (Fig 6B) where the level of the HAI-2 species with N-glycan branching also appeared to be slightly elevated (Fig 6C). Under permeabilizing conditions, the HAI-2 species with N-glycan branching was detected throughout the cells with some accumulation in vesicles with a tendency to be located preferentially on one side of the nuclei (Fig 6D). These data suggest that the HAI-2 species with N-glycan branching is distributed both on the cell surface and in intracellular pools. Under permeabilizing conditions, the HAI-2 species without N-glycan branching was detected at the periphery of the cells (Fig 6E) suggesting that this species has a largely intracellularly localization with a small proportion being targeted to the cell surface.

The low levels of the HAI-2 species without N-glycan branching localized on the cell surface appears to be the reason for its minimal role in matriptase inhibition. Matriptase is constitutively activated in breast cancer cells and the activated matriptase is detected on the cell surface (Fig 6H) where it coincides with the HAI-2 species with N-glycan branching (Fig 6I). The spontaneous activation of matriptase zymogen appears to have a non-uniform distribution with some cells having much more activated matriptase than others (Fig 6H and 6I). In contrast, when the cells were transiently exposed to a pH 6.0 buffer, uniform high-level matriptase zymogen activation was observed in the breast cancer cells (Fig 6K and 6L).

Two putative N-glycosylation sites are present on HAI-2: Asn-57 within Kunitz domain 1 and Asn-94 between the two Kunitz domains. When human HAI-2 shed from MNK 45 gastric cancer cells was subjected to internal amino acid sequencing [1], the two putative N-glycosylation sites were present in the HAI-2 proteolytic fragments, sequenced by Edman degradation. Asn-94 was identified in one of the internal sequences, 88-xatvte*n*atgdlatsrnaadssvpsap-114 (the underlined and italicized n), suggesting that it is not glycosylated, since if N-glycan were attached to Asn-94, it would not have been identified by Edman degradation. In contrast, although Asn-57 is located within another internal peptide sequence, 43-vvgrxrasmprwwy*x*vtxgsxqlfvygg-70, (*x* for the unidentified Asn-57), the fact that the residue was not identified suggests that the asparagine (Asn) residue was modified (or occupied by N-glycan). Collectively, the N-glycosylation consensus sequences and the data from the Edman degradation analyses of the naturally occurring HAI-2 provide evidence for the role of the Asn-57 in the N-glycosylation of HAI-2.

**Fig 7:**
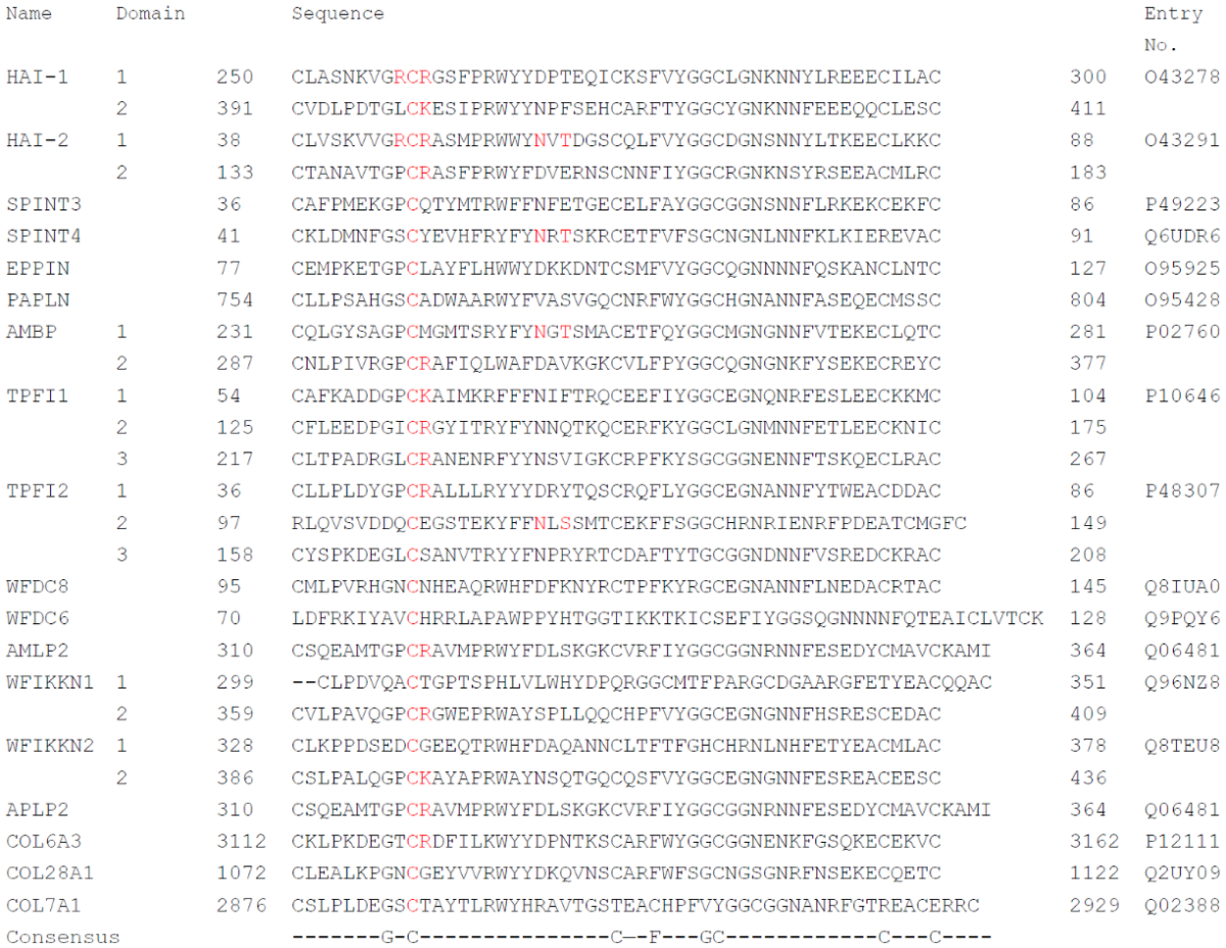
Alignment and comparison of the amino acid sequence of 27 Kunitz domains from 18 human proteins. The amino acid sequences of 27 Kunitz domains from 18 human proteins, including HAI-1, HAI-2, and bikunin (Alpha-1-microglobulin/bikunin precursor, AMBP) are compared. The P1 sites with Arg and Lys, and the P3/P4 sites with Arg, the second Cys residue and the putative N-glycosylation sites in the region corresponding to Asn-57 of HAI-2 are highlighted in red. The amino acid sequences were obtained from Uniprot (http://www.uniprot.org/). The names of these Kunitz proteins are provided on the left with their entry numbers on the right.

When comparing the amino acid residues flanking the equivalent of Asn-57 of HAI-2 with the sequence of other Kunitz domains-containing human proteins, only 4 out of 27 Kunitz domains from 18 human proteins retrieved from Uniprot (http://www.uniprot.org/) contain the putative N-glycosylation site at the corresponding position of Asn-57 in HAI-2 (Fig 7). These include Kunitz domain 1 of bikunin (Alpha-1-microglobulin/bikunin precursor, AMBP). Interestingly, this residue in bikunin, Asn-250 has been shown to indeed be occupied by biantanal N-glycan [24].

## Discussion

In the current study, we demonstrate that the Asn-57 residue of HAI-2 can be occupied by two different types of N-glycan: one with extensive terminal N-acetylglucosamine branching and the other likely to be an oligomannose type without terminal N-acetylglucosamine branching. In addition to altering the size and the rate of migration on SDS-PAGE, the two types of N-glycan appear to affect various aspects of HAI-2 biology such as the differential exposure of the epitopes recognized by the two HAI-2 mAbs, the subcellular localizations of the two HAI-2 species, and subsequently their role in the control of matriptase activity.

The co-expression of the different HAI-2 species at similar ratios in different cell lines raises the question of the relationship between the two HAI-2 species. The HAI-2 species undergo co-translational N-glycosylation by the addition of the 14-sugar glycan Glc3Man9GlcNAc2 to Asn-57 in the lumen of the ER, like other proteins that have N-glycan [25]. Prevention of the initial N-glycosylation by tunicamycin resulted in the expression of “naked” HAI-2 polypeptides detected at the same size by the two mAbs, suggesting that the two HAI-2 species, as defined by the two mAbs DC16 and XY9 are likely derived from the same HAI-2 transcript. When entering the *cis* Golgi, the HAI-2 N-glycan, like other N-glycosylated proteins, contains 8 terminal mannoses, many of which remain before entering the *medial* Golgi. The first and second branches of the N-glycan are generated in the *medial*-Golgi through the removal of mannose residues by α-mannosidases and the transfer of N-acetylglucosamine through the action of acetylglucosaminyltransferase. The suppression of levels of the 40- and 30-kDa HAI-2 forms along with the increased levels of 25-kDa HAI-2 by treatment with the α-mannosidase II inhibitor swainsonine suggests that a proportion of 25-kDa HAI-2 continues through the processing of N-glycan branching into the 30- and 40-kDa form which exits the Golgi apparatus into the secretory vesicles/granules. The remainder of the 25-kDa HAI-2 likely remains in the ER. The different subcellular localization of 30- and 40-kDa HAI-2 in the vesicles/granules and the perinuclear location of 25-kDa HAI-2 supports the hypothesis that 25-kDa HAI-2 is the precursor of the mature 30- and 40-kDa HAI-2. The N-glycan of 25-kDa HAI-2 containing terminal mannoses but no terminal N-acetylglucosamine also provides support for the hypothesis that 25-kDa HAI-2 is an immature species. Similarly, the observation that the N-glycan of 30- and 40-kDa HAI-2 contains terminal N-acetylglucosamine but no terminal mannoses supports the notion that these HAI-2 species have transited through the Golgi apparatus and completed N-glycosylation. This hypothesis is further supported by the fact that the shed HAI-2 species in the conditioned medium of breast cancer cells is the 30- and 40-kDa HAI-2 form with N-glycan branching and not the 25-kDa HAI-2 form without N-glycan branching.

The comparison of the immunofluorescent staining for the 25-kDa HAI-2 form in breast cancer cells under non-permeabilizing and permeabilizing conditions indicates that the vast majority of the 25-kDa HAI-2 for is intracellularly localized beneath the plasma membrane (Fig 6E). Interestingly, while at very low levels, 25-kDa HAI-2 was also clearly detected on the extracellular face of plasma membrane (Fig 6B). These data suggest that 25-kDa HAI-2 might have trafficked through the secretory pathway and reach the plasma membrane primarily on the cytoplasmic side. A small proportion of the HAI-2 species was translocated to the extracellular face of the plasma membrane of breast cancer cells. In breast cancer cells, 25-kDa HAI-2 may, therefore, function as a mature form with its own destination and target proteases, which do not include matriptase due to their different subcellular localizations. Furthermore, the high levels of 25-kDa HAI-2 and the relatively constant ratio of the 25-kDa form relative to the 30- and 40-kDa HAI-2 forms in a variety of cell lines supports the idea that 25-kDa HAI-2 might in fact be an independent entity rather than a precursor and/or intermediate of N-glycan branching in HAI-2-expressing cells not limited to breast cancer cells. It remains unclear how the cells allow a proportion of the 25-kDa HAI-2 to transit through the Golgi while at the same time N-glycan branching occurs on another proportion. The widespread expression of HAI-2 in conjunction with its aberrant subcellular localization in breast and other cancer cells not only provides those cancer cells with a new mechanism to control their enhanced proteolysis, but also provide a novel distinction between normal and cancer cells that might be exploited for the development of clinical applications. The identification of the target proteases that interact with the 25-kDa form of HAI-2 may reveal those proteases as potential targets for anti-cancer therapeutics, and the expression and aberrant subcellular localization of the two HAI-2 forms might have potential as cancer biomarkers.

HAI-2, the gene product of *SPINT 2*, was independently purified, cloned and designated as placental bikunin (PB) due to its containing two Kunitz domains and its high level expression in placenta [2]. The names HAI-2/PB reflect the functionality and protein domain structure of this Kunitz inhibitor which is related to HAI-1 and bikunin. The almost identical reactive center loops of Kunitz domain 1 in HAI-1 and HAI-2 are not only unique among the 18 human Kunitz-containing proteins (Fig 7) but also confer on HAI-1 and HAI-2 their inhibitory potency and specificity to HGF activator, matriptase and prostasin observed in solution. The functional relationship between the HAIs and matriptase in cell systems and *in vivo* is, however, dependent on factors beyond the simple biochemical properties of the proteins. Subcellular localization could play an even more important role in determining the degree to which matriptase is differently inhibited by HAI-1 versus HAI-2 [9]. In the current study, subcellular localization could also contribute to the differential role in matriptase inhibition between the two HAI-2 species, if one assumes that both HAI-2 species exhibit similar inhibitory potency against matriptase. In terms of protein domain structure, HAI-2/PB might be more closely related to bikunin than HAI-1. HAI-1 possesses an LDL receptor class A domain and a MANEC domain, neither of which are present in HAI-2. Although HAI-2 is highly related to HAI-1 in terms of the inhibitory specificity of their Kunitz domain 1s, HAI-2 resembles bikunin more in overall protein structure in that they only have two Kunitz domains, except for the presence of a transmembrane domain in HAI-2. Furthermore, HAI-2 also resembles bikunin with respect to the attachment of N-glycan to their respective Kunitz domain 1s. Among the 27 Kunitz domains in the 18 Kunitz-containing proteins, only 4 contain the putative N-glycosylation site at the end of the first beta-sheet in the Kunitz domain.

In summary, two HAI-2 species have been identified and characterized using the two mAbs DC16 and XY9, both of which recognize epitopes that reside on the HAI-2 polypeptide. The exposure of these two epitopes is significantly affected by the N-glycans attached to Asn-57, which defines the two HAI-2 species. The HAI-2 species with N-glycan branching appears to be mature HAI-2, which is intracellularly localized most likely in the secretory vesicles/granules in mammary epithelial cells and a proportion of which is translocated to the extracellular face of the plasma membrane where the HAI-2 species can inhibit matriptase in breast cancer cells. The second HAI-2 species bearing N-glycan without N-glycan branching has been identified by the mAb XY9. While the N-glycan is likely small in size, it significantly masks the epitope recognized by the mAb DC16. This lightly glycosylated HAI-2 species appears to be the premature product of N-glycosylation but a proportion of which is still able to transit through the secretory pathway and function as an independent entity in breast cancer cells. The identification and characterization of these HAI-2 species serves as a foundation to future study and understand of the pathophysiological role of this membrane-bound Kunitz inhibitor.
